# Comprehensive genomic analysis of type VI secretion system diversity and associated proteins in Serratia

**DOI:** 10.1099/mgen.0.001424

**Published:** 2025-06-13

**Authors:** Lili Jiang, Wenjing Yi, Yuzhe Zhao, Ning Zhu, Di Zhao, Zhihan Peng, Lei Song, Tingting Dong, Xin Jiang, Dandan Liu, Xufeng Ji, Qingtian Guan, Hongyu Jiang

**Affiliations:** 1 Health Examination Center, The First Hospital of Jilin University, Changchun, PR China; 2 Bioinformatics Laboratory, Center for Infectious Diseases and Pathogen Biology, The First Hospital of Jilin University, Changchun, PR China; 3 Department of Respiratory Medicine, Center for Infectious Diseases and Pathogen Biology, Key Laboratory of Organ Regeneration and Transplantation of the Ministry of Education, State Key Laboratory for Zoonotic Diseases, The First Hospital of Jilin University, Changchun, PR China; 4 Department of Laboratory Medicine, The First Hospital of Jilin University, Changchun, PR China

**Keywords:** comparative genomics, effector proteins, *Serratia *spp., type VI secretion system (T6SS)

## Abstract

The type VI secretion system (T6SS) is a contact-dependent contractile nanomachine widely distributed among Gram-negative bacteria, essential for interbacterial competition, host interactions and environmental adaptation. The T6SS encoded by *Serratia* is known to play roles in bacterial competition and contribute to niche adaptation. Yet, the diversity and distribution of T6SS loci and relevant proteins across different *Serratia* species remain underexplored. In this study, we conducted bioinformatic and comparative genomic analyses of the T6SS to expand our understanding of T6SS in *Serratia* and their distribution within the genus. A dataset comprising 2,337 *Serratia* genomes was analysed, identifying 4 distinct T6SS loci classified into 3 main types based on TssB sequence variation, with varying distributions across specific *Serratia* haplogroups. T6SS^serratia-1b^ subtype was found to be the most prevalent. By integrating homology searches and positional information, we expanded the known database of T6SS proteins in *Serratia*, identifying a range of effectors likely involved in interbacterial interactions and host adaptation. Additionally, statistical analysis reveals a strong correlation between the presence of T6SS and the diversity of associated genes, including not only known T6SS effectors and immunity proteins but also other co-occurring gene families, enabling the identification of multiple candidate loci potentially involved in bacterial competition and pathogenicity. These findings shed light on the complex distribution, evolutionary dynamics and functional diversity of T6SS and associated proteins in *Serratia*, advancing our understanding of their role in bacterial competition and environmental adaptation.

**Impact Statement**

*Serratia* spp. are widely distributed across various environments, including opportunistic pathogens and commensals in clinical and natural settings. It utilizes the type VI secretion system (T6SS) for interbacterial competition and ecological niche conquest. *In silico*, by employing genomic approaches, we identified 4 distinct T6SS locus subtypes and significantly expanded the repertoire of T6SS predicted effectors, identifying 69 effector families, including 58 novel ones. A novel statistical and comparative genomic method was developed to predict T6SS-associated proteins based on their co-occurrence with complete T6SS loci, providing a valuable tool for future research. Future investigations into the T6SS of *Serratia* spp. and their secreted effectors will deepen our understanding of their role in bacterial competition and adaptation.

## Data Summary

All genomic data used in this study were obtained from publicly accessible databases. Detailed information, including accession numbers for each genome, is provided in Table S1. We share the Python scripts used in this study via GitHub (https://github.com/Guan-biolab/Serratia_T6SS).

## Introduction

The genus *Serratia* is a member of the *Yersiniaceae* and is considered the opportunistic human, plant and insect pathogens widely distributed in soil, water, plants, animals and invertebrates [1]. Currently, there are 36 species within the genus *Serratia*, 23 of which are published with accepted names [List of Prokaryotic Names with Standing in Nomenclature (accessed: 3 January 2023); https://lpsn.dsmz.de/genus/serratia]. Although not all species are pathogenic, some have become important pathogens in hospitals, *Serratia marcescens* being the most commonly isolated, with strong pathogenicity and wide drug resistance. *S. marcescen*s causes meningitis [2,3], urinary tract infections [4], pneumonia and other respiratory diseases [5], bloodstream infections [6,7], endocarditis [8] and different types of wound infections [9]. While some *Serratia* strains are harmless commensals, others exhibit pathogenic potential, highlighting the genomic complexity of *Serratia* species and the need for further research to elucidate their impact on human health. Bacteria have evolved various secretion systems throughout their life cycles and during infection, enabling them to adapt to different environments and interact with hosts. However, systematic analyses of the type VI secretion system (T6SS) among *Serratia* species remain limited.

To date, 11 distinct secretion systems have been identified in bacteria, ranging from the type I secretion system to the type XI secretion system [10,15]. Among these, the T6SS has been recognized as a critical virulence factor in pathogenic bacteria. The T6SS is a widely distributed protein secretion apparatus found in ~25% of Gram-negative bacterial species [16,17]. It functions as a contact-dependent cell-killing mechanism, allowing bacteria to compete with other micro-organisms and interact with host cells [18,19]. Advances in the T6SS understanding include the functional and structural characterization of 13 core components, which are thought to comprise the basic secretion apparatus [20,21]. Upon contact with host cells or competing bacteria, the TssBC sheath of the T6SS rapidly contracts, propelling the puncturing device, including the effector protein, through the basal complex, out of the cell and into an adjacent target cell. The tubular structure then penetrates the target cell membrane, injecting toxic effectors into neighbouring cells [22], causing cell death or stasis by various mechanisms [23,25]. Thus, T6SS is a key determinant of competitive ability and pathogenic potential. However, analyses of T6SS distribution and effector identification in *Serratia* spp. are limited. It has been reported that either two or four T6SSs in the genus *Serratia* and the functions of this locus have not been elucidated [26]. In this study, we identified four T6SS loci present in more than 2% of high-quality genomes at the level of *Serratia* spp. and further analysed the distribution of the locus.

To understand how the T6SS influences both eukaryotic and bacterial cells, it is crucial to identify the substrate proteins secreted by this system. T6SS effectors can be divided into structural secretion proteins (specialized proteins) and non-structural secretion proteins (cargo proteins) [27]. Cargo effectors are secreted by deploying an adaptor protein or binding non-covalently to the haemolysin-coregulated protein (Hcp/TssD), valine−glycine repeat protein G (VgrG/TssI) or proline−alanine−alanine−arginine (PAAR) subunits [28,29]. However, specialized effectors have an effector domain that is covalently attached to the C-terminal extended domains of a component of the extracellular puncturing device, such as the VgrG/Hcp/PAAR complex or PAAR-containing Rhs proteins. The VgrG and Hcp proteins were the first identified T6SS substrates secreted into the extracellular environment by T6SSs [30]. However, as core components of the T6SS machinery, Hcp and VgrG function primarily as structural elements of the secretion apparatus rather than as true secreted effector proteins.

Identifying T6SS effectors remains a significant challenge, as substrate recognition lacks conserved sequence motifs and T6SS expression is often repressed under standard laboratory conditions. Although comparative genomic approaches have been used to identify T6SS effectors in bacteria such as *Vibrio parahaemolyticus* [31], *Pseudomonas aeruginosa* [32,33] and *Salmonella* [34], which highlight the potential importance of T6SS inter- and intraspecific diversity in bacterial ecology and evolution. However, comprehensive studies at the *Serratia* genus level are still limited. To date, only 11 effectors have been identified from the *Serratia* genus, including small secreted protein (Ssp1-6), Rhs1/Rhs2, secreted lipase-like protein (Slp), type VI secretion ADP-ribosyltransferase effector 1 (Tre1) and the antifungal effectors (Tfe2) [35,38]. To address this, we adopted a comprehensive *in silico* strategy to systematically identify T6SS-associated proteins in *Serratia* spp., combining multiple complementary approaches: (1) a homology-based search to detect orthologues of known effectors across 2,337 high-quality *Serratia* genomes, (2) a domain-based scan for specialized effectors by identifying extended toxic domains fused to core structural proteins (Hcp, VgrG and PAAR) and (3) a genomic neighbourhood analysis to detect cargo effectors downstream of *vgrG*, including operon-based co-localization. This multi-layered approach provides a broader view of the diversity and distribution of potential T6SS-associated proteins in *Serratia*. As a result, we have identified 69 types of effector families with an orthologous search approach, not only including the 11 types of effector families that have been identified in *Serratia* but also expanding the repository with 58 effector families that have been shown as effectors in other species. Furthermore, we have also identified 432 potentially specialized effectors and 87 potential cargo effectors using their position information of genomic regions downstream of *vgrG*. Notably, 42 cargo effectors exhibited co-localization with VgrG in an operon.

Traditional approaches to identifying T6SS-associated proteins largely focused on known effectors and their homologues, mainly relying on positional information and experimental validation. However, these strategies are often limited by incomplete functional annotations, unclear secretion signals [35] and the context-dependent expression of T6SS under laboratory conditions [36]. In this study, we develop a generalized method for identifying not only antibacterial T6SS effectors but also other T6SS-associated proteins. We employed a comparative genomic approach to identify orthologous groups significantly associated with T6SS presence in *Serratia* spp. We calculated normalized T6SS+/T6SS− protein ratios and applied a chi-squared test to assess the significance, creating a statistically robust method to identify T6SS-associated orthologues. Additionally, 95 T6SS-associated orthologous groups (TAOGs) predicted as effectors showed a significant distribution in the presence/absence of T6SS. These findings have extensively enlarged our understanding of T6SS effectors, especially in *Serratia*. This work significantly expands our understanding of previously unknown T6SS-associated proteins and provides ideas for exploring new effectors in *Serratia* spp.

In summary, our study provides a comprehensive genomic perspective on the diversity, organization and functional potential of the T6SS and its associated proteins in *Serratia*, serving as a foundation for future research into its biological significance.

## Methods

### Data acquisition and analytical workflow

A total of 3,285 genomes, classified as *Serratia* spp., were obtained from the NCBI GenBank database on 3 January 2024 (Table S1, available in the online Supplementary Material). The workflow analysis we employed for T6SS locus identification, T6SS effectors, immunity orthologue characterization, visualization and TAOG prediction is depicted in Fig. S1. Overall, the research pipeline consists of four sections (Fig. S1): (1) data acquisition and quality control, (2) T6SS locus identification and characterization, (3) quantification and distribution of known T6SS-related proteins and (4) identification of TAOGs. The details of the methods are described in the following sections.

### Identification and classification of T6SS loci

A total of 2,337 high-quality genomes from 22 *Serratia* species were obtained after the qualification filtration. The 2,337 *Serratia* genomes were annotated by Prokka (v1.12) [39]. The resulting *Serratia* high-quality genome annotation results were aligned with the structural core components from the SecReT6 [40] database using Proteinortho (v6.3.1) [41]. T6SS loci were identified based on 13 T6SS core components (TssA-M) in the database, requiring a maximum of 10 non-core genes between 2 genes to be within the same locus. Occurrences of the same arrangement order exceeding 2% of the total genomes were counted as a locus arrangement. The complete T6SS cluster (with 13 T6SS different core components, T6SS+), partially T6SS (with 5–12 different T6SS core components) and non-T6SS (with 0–4 different T6SS core components, T6SS−) were defined by the number of core genes in each locus. Four subtypes of T6SS clusters were identified among all strains containing the complete T6SS clusters in *Serratia*, based on the order of the core proteins in the locus.

In the meantime, we also classified the loci using the classical TssB typing method [40]. In this study, the T6SS loci were classified and displayed in subtypes based on experimentally validated TssB proteins from the SecReT6 database [40]. To classify the T6SS gene locus of *Serratia* spp., the CD-HIT tool (v4.8.1) [42] (with a sequence identity threshold of 1 and a length difference cutoff of 1) was used to cluster sequences of the 3,045 TssB proteins from complete T6SS systems. A total of 112 TssB unique protein sequences were obtained and combined with the validated TssB sequence data for phylogenetic reconstruction. The phylogenetic tree was then constructed using a step-by-step approach: TssB sequence alignment was performed with MAFFT (v7.520) [43], followed by sequence trimming using trimAl (v1.4.rev15) [44]. The phylogenetic tree was then constructed using IQ-TREE (v2.2.6) [45], with 10,000 bootstrap replicates, and the maximum-likelihood method was employed to infer evolutionary relationships. The resulting phylogenetic trees were visualized and annotated using iTOL (v6) [46] to represent the distribution of T6SS clusters and TssB types. According to the results of TssB types, the identified T6SS loci were classified into three main types: T6SS^serratia-1^ (type i3), which includes two subtypes, T6SS^serratia-1a^ and T6SS^serratia-1b^, based on the difference in core gene arrangement; T6SS^serratia-2^ (type i1); and T6SS^serratia-3^ (type i2). The T6SS loci were visualized based on the size and location of the genes using the gggenes (v0.5.1) package.

In this study, BLASTN (v2.15.0+) [47] was utilized to assess the occurrence of horizontal gene transfer (HGT) at four subtypes of T6SSs in *Serratia*. The nucleotide (NT) sequences of the four T6SSs were aligned with corresponding sequences from non-*Serratia* species, and the resulting data were visualized using Easyfig (v2.2.5) [48].

### Distribution and typing of T6SS loci in *Serratia* spp.

To select phylogenetically representative genomes and avoid outlier strains, average nucleotide identity (ANI) analyses were performed using fastANI (v1.32) [49], and strains were clustered by hierarchical clustering based on an all-to-all ANI matrix with a minimum number of pairwise distances within nodes (threshold 0.03). The strain with the highest degree of integrity was selected as the representative genome from each species and was used to construct the phylogenetic tree of the genus *Serratia*. The reference genome of *Yersinia pestis* (accession number GCA_033786385.1) served as the outgroup. The multiple sequence alignment file generated by PhyloPhlAn3 (v3.0.68) [50] was used for phylogenetic tree construction. A total of 344 conserved genes were selected from the 23 genomes for alignment and tree generation. To enhance the robustness of the results, IQ-TREE [45] was employed to perform 10,000 bootstrap replicates, utilizing the maximum likelihood method to infer evolutionary relationships [45].

### Identification of specialized effectors in the *Serratia* genus

The Hcp, VgrG, PAAR and PAAR-like domain hidden Markov model (HMM) profiles were constructed using hmmbuild, based on representative seed sequences from the Pfam database. These profiles were then used to identify matching proteins using hmmsearch (HMMER v3.4) [51]. Genes containing Hcp, VgrG, PAAR and PAAR-like Pfam domains by using InterProScan (v5.73-104.0) [52] were extracted from the *Serratia* genus, which include genes with ‘core’ (no C-terminal extensions) T6SS domains, as well as those that match the architecture of specialized T6SS effectors (i.e. those with a C-terminal extension), with a total of 16,017 protein sequences (Table S2). We followed the method described by Geller *et al*. [53], in which sequences with C-terminal extensions large enough to encode a domain were filtered, with boundaries defined by the end coordinates of the alignments to core T6SS domains in each gene. For PAAR and Hcp, the C-terminal domains with ≥120 AA after the end of the PAAR or Hcp domain were filtered. For VgrG domains in the *Serratia* genus, we initially selected those with an overall length greater than 536 AA, based on the mean length of the core VgrG (no C-terminal extension) distribution (509 AA), and added one SD (27 AA). For these VgrG domains, which include DUF2345-like needle domains and gp5 C-terminal trimerization domains, the end coordinate of VgrG was redefined using these domains, and those with extensions of less than 120 AA were removed.

### Identification and annotation of VgrG-associated putative effector proteins

It is widely recognized that effector and immunity proteins are located downstream of the *vgrG* tip protein [54,56]. To identify potential *vgrG*-associated effectors in *Serratia*, we have identified the proteins encoded downstream of *vgrG*. Based on the VgrG domain identified using the above HMM method, we further identified three proteins downstream of *vgrG*. The obtained protein sequences were further annotated by orthologous search with T6SS component proteins, effector (T6SEs) and immunity (T6SIs) of the SecReT6 repository using Proteinortho [41] (Table S2), and proteins annotated as component proteins and immunity proteins were excluded from the effector pool. If a protein has multiple functional annotations, its classification was manually determined. An in-depth functional domain analysis of the protein sequences was performed with the Conserved Domain Database (CDD) [57] to study the domains associated with the effectors. The visualization was performed to analyse the relationships of the effectors within the potential effectors using ChiPlot. Operon-mapper (https://biocomputo.ibt.unam.mx/operon_mapper/) was used to analyse whether the predicted putative cargo effectors downstream of VgrG were located within the same operon as VgrG.

### Distribution patterns of T6SS-associated orthologous groups in T6SS^serratia-1b^+ and T6SS^serratia-1b^− conditions in *Serratia*

The number of T6SS types is not uniformly distributed in the *Serratia* genus, with T6SS^serratia-1b^ being the most abundant one, appearing in 2,116 instances across the genus (T6SS^serratia-1a^ appears 770 times, T6SS^serratia-2^ 68 times and T6SS^serratia-3^ 86 times). To avoid skewing the statistical analysis due to the dominance of T6SS^serratia-1b^ over other types, we excluded the other T6SS types and focused our study on T6SS^serratia-1b^.

All known effectors, immunity proteins and regulator proteins within the genus *Serratia* were identified using the SecReT6 database. Homologous proteins were then compared using Proteinortho software, and the number of homologous protein clusters was counted. The genomes were subsequently divided into two groups (T6SS^serratia-1b^+ and T6SS^serratia-1b^−) based on the integrity of the T6SS^serratia-1b^ locus, and partial T6SS loci were excluded from the analysis. We analysed the relationship between known effectors, immune and regulatory proteins and the presence of T6SS. We identified and qualified the effector, immune and regulatory protein clusters in the T6SS^serratia-1b^+ and T6SS^serratia-1b^− genomes. Subsequently, a *t*-test was conducted to assess whether there were any differences in the number of known T6SS-relevant proteins between the T6SS^serratia-1b^+ and T6SS^serratia-1b^− genomes. Finally, we calculated the normalized ratio of T6SS^serratia-1b^+ to T6SS^serratia-1b^− genomes for each homologous protein and plotted bar graphs to represent this quantitative relationship.

### Identification of T6SS^serratia-1b^-associated orthologous groups

We aim to expand our investigations to identify a broader family of T6SS-associated proteins. We investigated the distribution of T6SS-relevant proteins in contexts where the T6SS^serratia-1b^ locus is either present or absent. Protein clustering was carried out on 2,337 genomes in 22 *Serratia* species using CD-HIT [42] (with a sequence identity threshold of 0.7 and a length difference cutoff of 0.7). This process eliminated redundant similar sequences and identified homologous sequences, and a total of 103,055 clusters were identified. We looped through all homologous protein families present in strains containing the T6SS^serratia-1b^ cluster and normalized the T6SS+ and T6SS− strains of genome counts in each protein family. A chi-square test was employed to statistically evaluate the normalized data, allowing for the identification of protein families significantly associated with the presence of the T6SS^serratia-1b^ locus. Then, we selected representative top 500 clusters (T6SS^serratia-1b^+/ T6SS^serratia-1b^−: log2 presence/absence value >3.88, *P*<0.001, top 500 clusters, Table S3), and these clusters were mapped back to each genome and arranged in the order of gene positions, connecting them and visualized using Cytoscape (v3.10.1) [58]. Furthermore, the top 500 T6SS-associated proteins were functionally annotated using Proteinortho [41] with SecReT6, Bastion6 [59], Pfamscan (v) [60] and eggNOG-mapper (v2.1.12) [61], respectively (Table S3).

The formula for the log2 presence/absence value is as follows:


$$\log_{2}\left(\frac{Presence\;genomes\;count\times T6SS\;absence\;genomes/(T6SS\;presence\;genomes+1)}{Absence\;genomes\;count+1}\right)$$

To test our hypothesis, we manually selected three Bastion6 predicted effectors for preliminary analysis. We used AlphaFold2 (v2.3.1) [62] to predict the 3D structures of three putative effector proteins and the effectors from the SecReT6 database. We then used Foldseek (v9.427df8a) [63] to compare the predicted structures of these potential effectors with those in the SecReT6 effector database based on structural similarity. Additionally, we performed a Position-Specific Iterated blast (PSI-BLAST v2.15.0+) [64] analysis to investigate the distribution of these effectors across various bacterial species.

## Results

### General characteristics of T6SS loci in *Serratia*

**Fig. 1.**
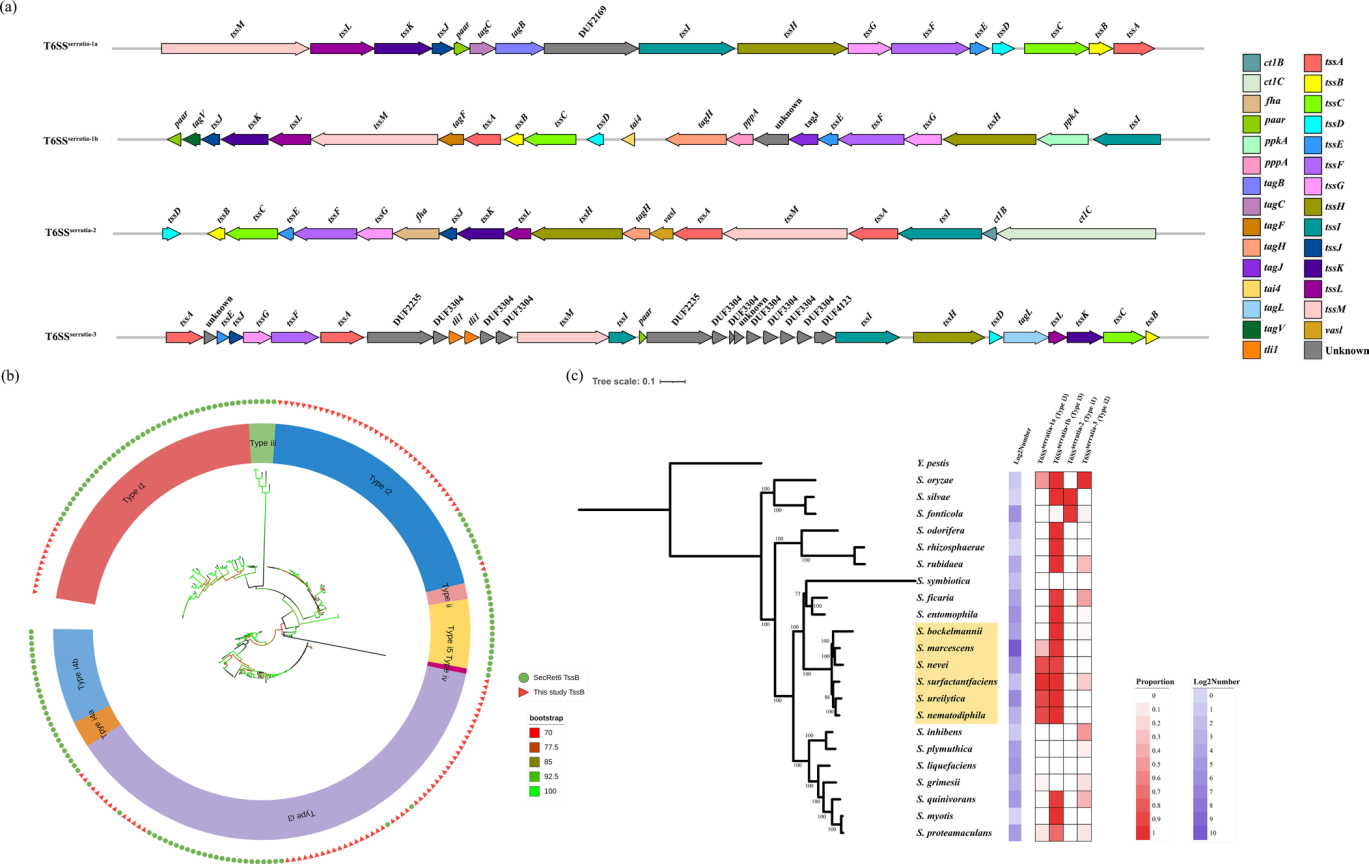
Characterization, classification and phylogenetic distribution of T6SS loci in *Serratia* spp. (**a**) Schematic showing the four identified T6SS loci in *Serratia*. Different colors denote distinct genes or structural domains. Arrows indicate the transcription direction of each gene. (**b**) Classification of T6SS loci in the genus *Serratia* using SecReT6 database TssB typing. The T6SS in *Serratia* can be categorized into three main types based on TssB protein typing, and these loci are classified as T6SS^serratia-1a^ (type i3), T6SS^serratia-1b^ (type i3), T6SS^serratia-2^ (type i1) and T6SS^serratia-3^ (type i2). (**c**) Phylogenetic tree of 22 *Serratia* species. The maximum-likelihood phylogenetic tree of 22 *Serratia* species was constructed using PhyloPhlAn3 with whole-genome sequences and *Y. pestis* as the outgroup. The tree was generated with 10,000 ultrafast bootstrap replicates. Highlighted species (in yellow) represent a distinct clade enriched in T6SS clusters. Annotations are provided to the right of the figure. The left section displays the log2 counts of genomes for each species, while the right section illustrates the percentage of the four T6SS clusters present in each species on the tree. Darker cells represent higher values, whereas lighter cells indicate lower values.

A total of 2,337 high-quality genomes from the NCBI were obtained through strict filters such as genome integrity and contamination level, representing strains collected from 50 countries and regions with a span from 1819 to 2023. We investigated the prevalence and distribution of T6SS clusters in complete genomes of *Serratia* spp. strains using the workflow as described (Fig. S1). Our analysis identified four T6SS loci. Meanwhile, we typed the T6SS loci based on TssB types [40]. The 3,367 TssB protein sequences were identified in 2,337 genomes, with 3,045 present in complete T6SS loci. The 322 TssB sequences that were not identified within the T6SS locus are likely caused by the fragmentation of certain assemblies. T6SSs can be classified into four types (i, ii, iii and iv), and T6SSi consists of six subtypes (i1, i2, i3, i4a, i4b and i5) based on TssB typing [65,66], of which T6SSi is the most common type found in *Serratia*. Based on the classification of TssB types, the four loci are named T6SS^serratia-1a^, T6SS^serratia-1b^, T6SS^serratia-2^ and T6SS^serratia-3^ (Fig. 1a) [26,67, 68]. Among them, T6SS^serratia-2^ and T6SS^serratia-3^ were less frequently observed in the *Serratia* genus. We discovered that T6SS^serratia-1a^ and T6SS^serratia-1b^ are both defined as type i3, T6SS^serratia-2^ as type i1 and T6SS^serratia-3^ as type i2 (Fig. 1b), which reflects the robustness of the TssB typing and the diversity of T6SS loci within the genus. In conclusion, the T6SS clusters identified in *Serratia* spp. belonged to either subtype i1, i2 or i3 (Fig. 1b), with most *Serratia* species containing two subtypes of T6SS (Fig. 1c).

### Distribution of T6SS loci across the *Serratia* genus

We inferred the genus phylogeny from the whole-genome data using representative genomes of 22 species of the *Serratia* genus (Fig. 1c). It is evident that there are distinct branches evolved in this phylogeny, and different haplogroups are formed, each with a varying number of T6SS clusters, except *Serratia symbiotica* that has no T6SS locus, which is likely because only four assemblies are available for the species. However, the highlight haplogroup, which contains *Serratia ureilytica*, *Serratia surfactantfaciens*, *Serratia nematodiphila*, *Serratia nevei*, *Serratia bockelmannii* and *S. marcescens*, stands out with a significantly higher number of T6SS clusters, especially T6SS^serratia-1b^, than the others. *S. marcescens* species shows the highest prevalence of T6SS^serratia-1a^, T6SS^serratia-1b^ and T6SS^serratia-3^, with proportions of 33.83%, 98.33% and 1.72%, respectively. It is worth noting that this haplogroup species contains at least two T6SS clusters, in which *S. marcescens* is a clinically important opportunistic pathogen (Fig. 1c).

**Fig. 2.**
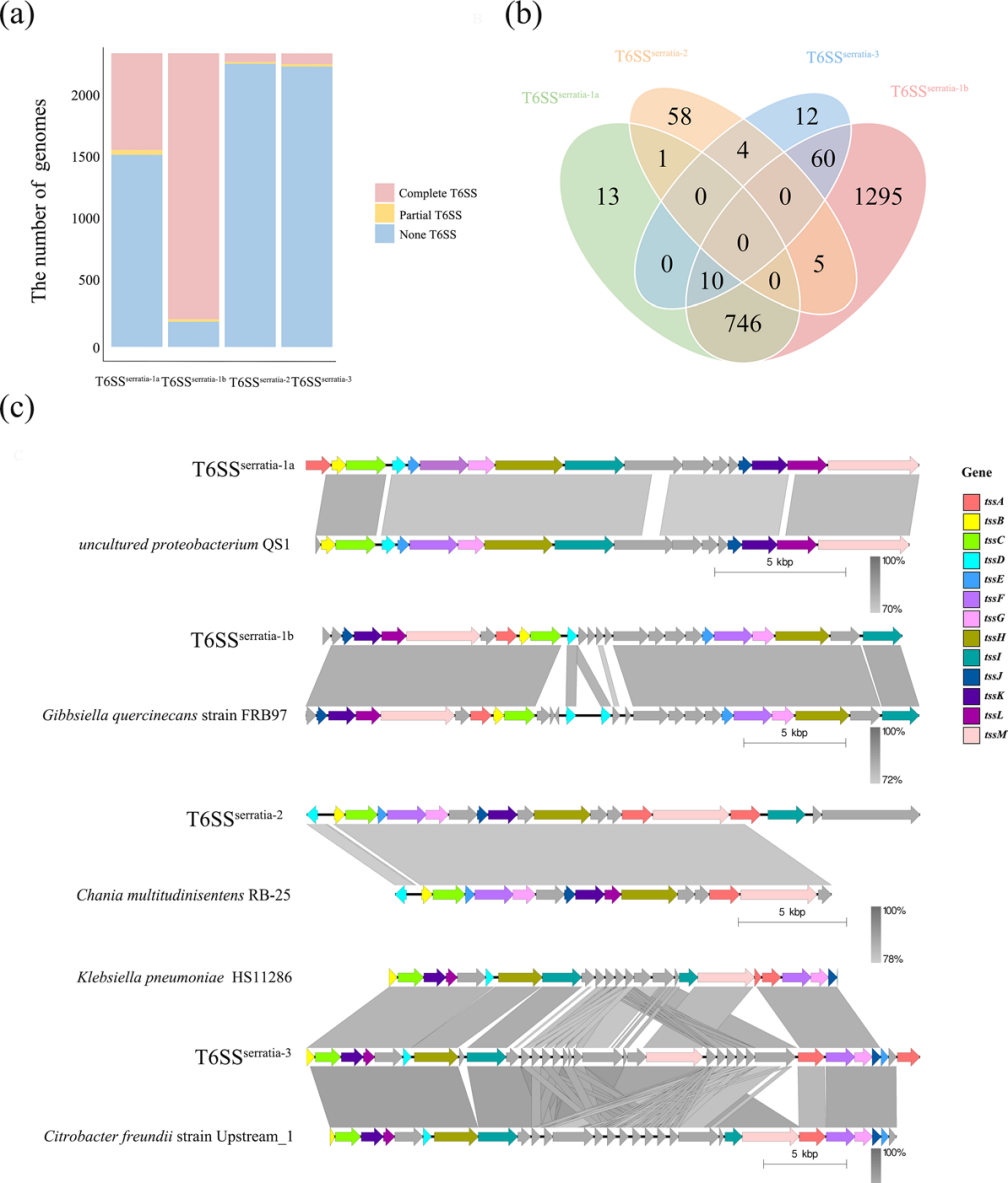
Characterization of T6SS loci in *Serratia* spp. (**a**) The distribution of T6SS loci across *Serratia* spp. classified based on core gene counts: a complete T6SS contains ≥13 structural genes, a partial T6SS includes 5 to 12 core genes and a non-T6SS contains <5 core genes. (**b**) Venn diagram illustrating the distribution of the four T6SS subtypes across different *Serratia* strains. (**c**) Syntenic and NT comparisons of the four T6SS subtypes in *Serratia* with related species suggest possible HGT events.

The data from this study showed a high number of T6SS^serratia-1a^ and T6SS^serratia-1b^, with 770 and 2,116 instances, respectively. In contrast, T6SS^serratia-2^ and T6SS^serratia-3^ were present in a relatively smaller number of 68 and 86 (Fig. 2a). T6SS^serratia-1a^ and T6SS^serratia-1b^ show the most significant overlap with 756 shared strains. There is also a smaller fraction of co-occurrence of different loci. For example, a total of ten strains contain T6SS^serratia-1a^, T6SS^serratia-1b^ and T6SS^serratia-3^, five strains shared by T6SS^serratia-1b^ and T6SS^serratia-2^ and a single strain contains both T6SS^serratia-1a^ and T6SS^serratia-2^. No strains were shared among the four T6SS locus subtypes (Fig. 2b). We analysed the distribution of the four subtypes of loci in different strains using Fisher’s exact test. The presence of T6SS^serratia-1a^ was found to be correlated with T6SS^serratia-1b^ (Fisher’s test, *P*<2.2e-16), whereas no such correlation was detected among the other loci (Fisher’s test, *P*>0.05) (Table S4).

The four T6SS loci in *Serratia* exhibit high homology across different species. The closest matching strains were identified as *uncultured proteobacterium* QS1, *Gibbsiella quercinecans* strains FRB97, *Chania multitudinisentens* RB-25 and *Citrobacter freundii* strain Upstream_1, respectively (Fig. 2c and Table S5). This similarity indicates that HGT may have played a role in the distribution of these T6SS types among *Serratia* and related species.

### Homology-based identification and diversity analysis of T6SS effectors and immunity proteins in *Serratia*

**Fig. 3.**
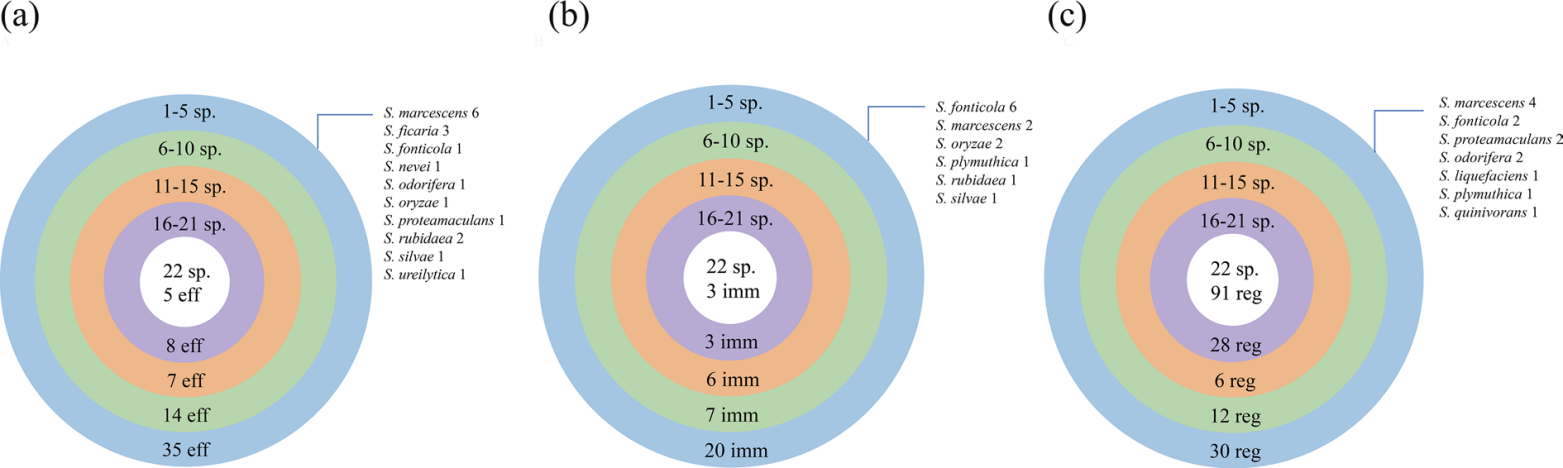
The distribution of effectors, immunity proteins and regulators in species of the *Serratia* spp. The circle diagram illustrates a collection of effector (eff) (**a**), immune (imm) (**b**) and regulator (reg) (**c**) proteins shared by different species (sp.) of *Serratia*. The top of each circle shows the number of species in which these proteins are found (sp.), while the bottom shows the number of proteins in the collection (eff, imm or reg). The labels in the upper right of each diagram indicate the distribution of proteins present in a single species. The numbers next to each species represent the number of unique T6SS-associated protein types found in that species.

*Serratia* spp. exhibit a high diversity of effector proteins. In this study, we have identified a total of 69 types of effector orthologues by utilizing a homologous search approach (Figs 3a and S2). In addition to the 11 effector families that have already been characterized in *Serratia*, Tre1 [69], Slp [38], Tfe2 [35], Rhs1 [70], Rhs2 [70], Ssp1 [71], Ssp2 [71], Ssp3 [35], Ssp4 [36], Ssp5 [36] and Ssp6 [37], we have also identified 58 orthologues that have been discovered in other species but have not yet characterized in *Serratia* such as Nte5 and Nte4 (Table S6). Notably, five effector families were identified in all species of the *Serratia* genus: EseM, Rhs1, Rhs2, AmpDh3 and ModA. Through the SecReT6 database and literature reports, we found that Rhs1 and Rhs2 in *Acinetobacter baumannii* shared identity to Rhs T6SS effector proteins in *P. aeruginosa* and *S. marcescens* [72]. However*,* these have not been studied in the *Serratia* genus before. The remaining 64 classes of effector families were distributed among different *Serratia* species. In addition, 18 distinct classes of T6SE families have been identified within a single species. Notably, *S. marcescens* harbours six unique effector families, including EFF01498 (Tpe), Hcp3, TseN and three variants of Tde (TseTBg1, Hcp-2 and TseTBg3). This diversity highlights the extensive functional repertoire of T6SEs within a single organism, suggesting a high degree of specialization and adaptation in interbacterial interactions. These findings illustrate the diversity of effector families in *Serratia* spp. (Fig. 3a). Furthermore, *Serratia* possesses a total of 39 immune protein groups (Fig. 3b), with 3 widely distributed across different species, which are Rhs2I, Hcp-ETI and TsiT. Additionally, there are 13 classes of immunity families distributed in a single species, and *Serratia fonticola* has 6 classes of immunity families, while *S. marcescens* has 2 classes. In the contract, the regulators in *Serratia* spp. are more uniformly present compared with effectors and immunity families (Fig. 3c), and most effector families (35/69) are present only in 1–5 *Serratia* spp. (Fig. 3a).

### VgrG-guided discovery of specialized and cargo effectors

**Fig. 4.**
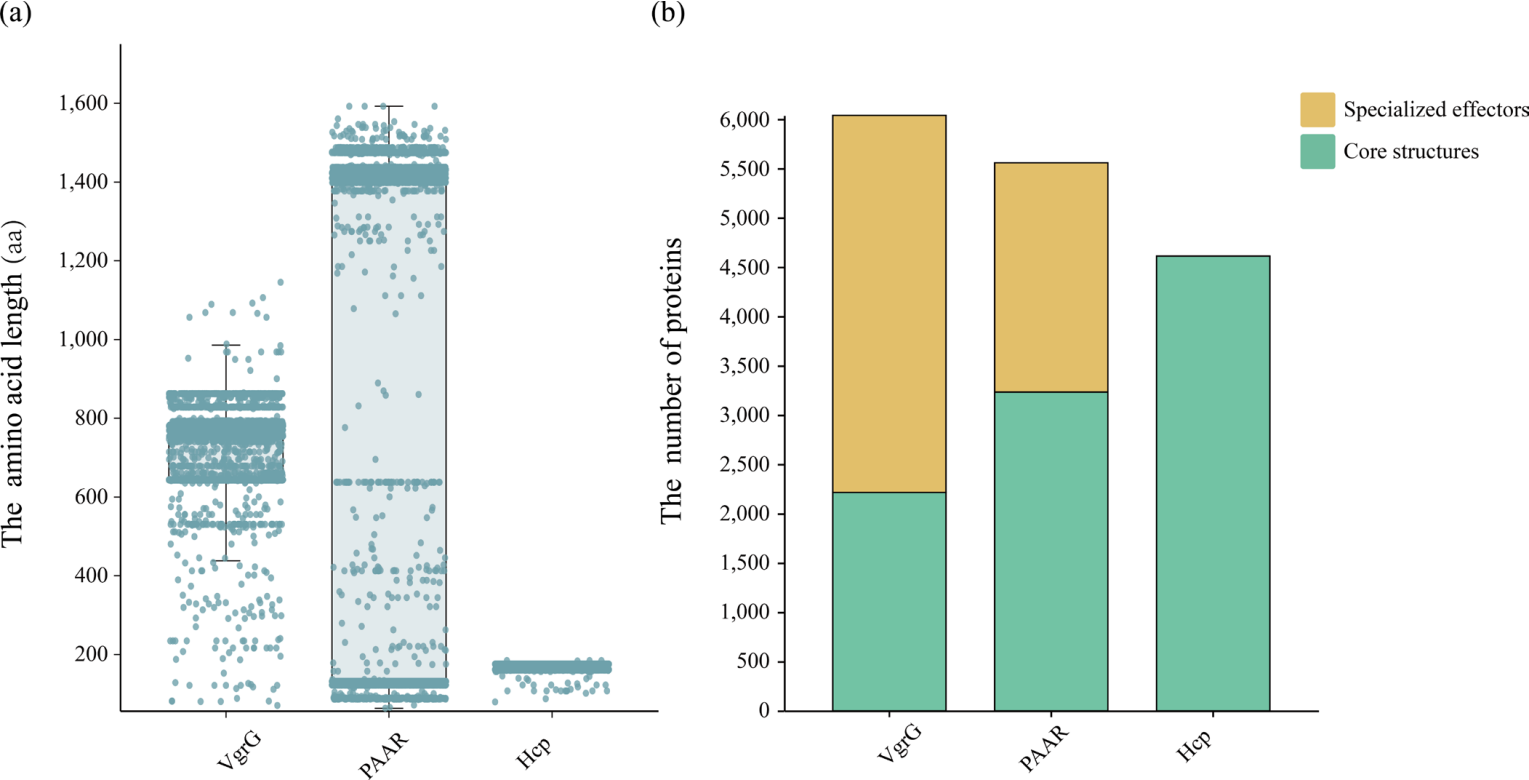
Characterization of VgrG, PAAR and Hcp in *Serratia.* (**a**) The AA length (aa) distribution of VgrG, PAAR and Hcp proteins across the *Serratia* genus. Each dot represents a single protein, with the length on the y-axis. (**b**) The proportion of core structural proteins and specialized effectors of VgrG, PAAR and Hcp proteins. VgrG and PAAR comprise core structures and a significant proportion of specialized effectors, whereas no specialized effectors were identified in Hcp.

Effectors are proteins secreted by secretion systems that enable bacteria to interact with other bacteria and the environment [73]. T6SS effectors can be classified into specialized (structural secretion proteins) and cargo (non-structural secretion proteins). Specialized effectors are structural elements of the T6SS with functional effects, including the VgrG/Hcp/PAAR complex, which contains distinctive C-terminal extensions conferring toxic activities [38]. Cargo effectors, on the other hand, are secreted by binding non-covalently to adaptor proteins or the Hcp, VgrG or PAAR subunits [38]. We analysed 6,038 VgrG, 5,556 PAAR and 4,611 Hcp proteins across the dataset to provide a global overview of effector specialization. This study identified 3,824 (3,824/6,038, 63.33%) VgrG and 2,324 (2,324/5,556, 41.83%) PAAR as possible specialized effectors, based on the presence of long C-terminal extensions. However, no specialized Hcp effectors were identified (Fig. 4 and Tables S7–S9)

**Fig. 5.**
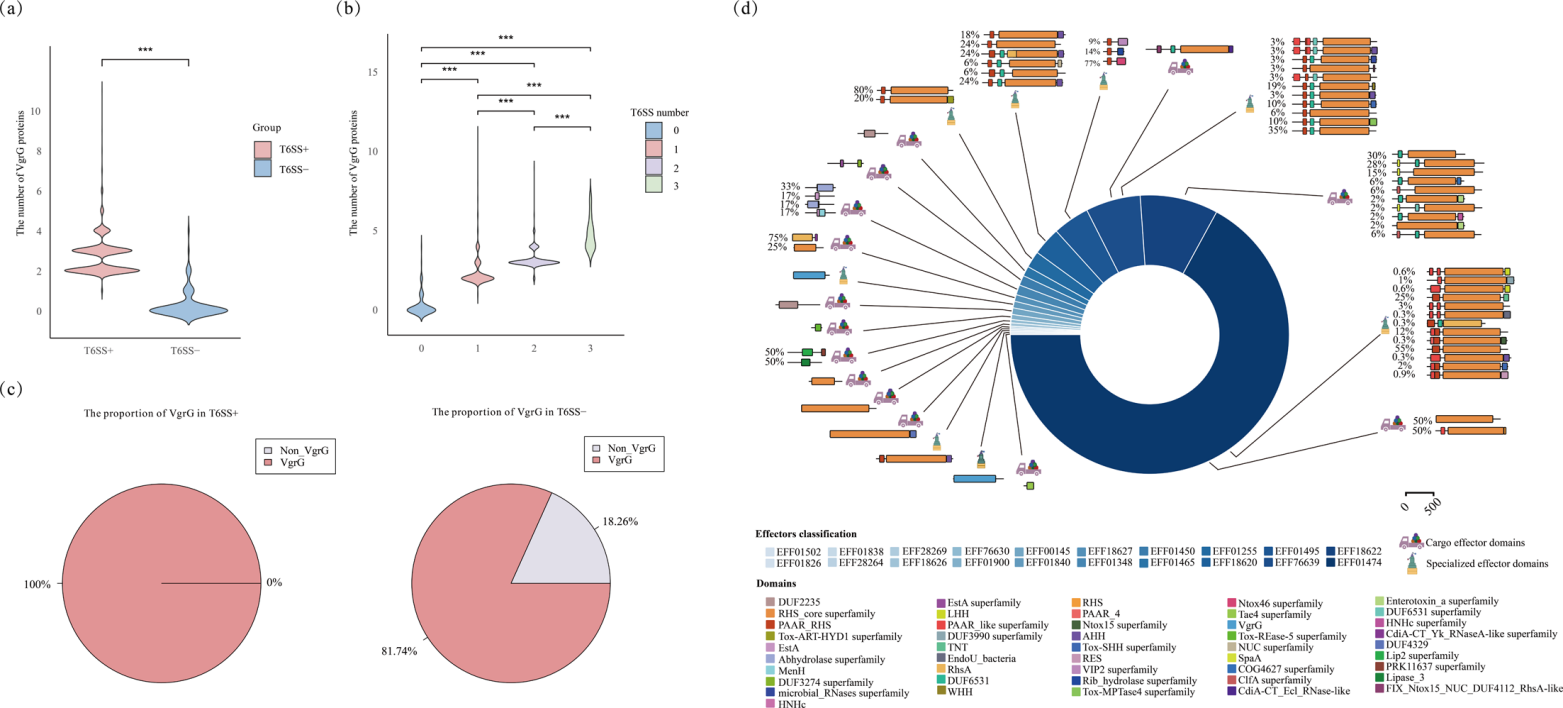
Distribution and functional annotation of VgrGs and downstream proteins in *Serratia*. (**a**) Distribution of VgrG protein counts in the *Serratia* genomes with and without T6SS. (**b**) Comparison of VgrG counts in *Serratia* genomes containing 0–3 T6SS. (**c**) The proportion of VgrG in T6SS+ and T6SS− genomes is illustrated by pie charts. (**d**) The architectures of the effectors identified in the three proteins downstream of VgrG. A total of 20 homologous protein families were identified. Each effector protein family is further classified as a specialized effector or a cargo effector. The percentage of particular effector architecture within each protein family is shown to the left of the architecture.

Previous studies revealed that T6SS effector and immunity proteins are frequently encoded in the VgrG neighbourhood [74,75]. The number of VgrG proteins in T6SS+ genomes was significantly higher than in T6SS− genomes (*P*<0.001) (Fig. 5a), and the quantity of VgrG proteins was proportional to the number of T6SSs (*P*<0.001) (Fig. 5b). All T6SS+ genomes contain at least one VgrG, while 18.26% of T6SS− genomes do not contain any (Fig. 5c).

Given the strong association between VgrG and effector diversity, we refined our homologue-based prediction by analysing downstream genes of *vgrG* in 2,337 *Serratia* genomes, classifying them as specialized or cargo effectors based on domain architecture and genomic context. We explored the diversity of the proteins downstream of *vgrG* in the 2,337 *Serratia* genomes. A total of 12,816 proteins (Table S2) were identified among the 3 proteins downstream of *vgrG* in 2,337 genomes within the same contig. Through the homology analysis of the 3 genes downstream of *vgrG*, we identified 519 proteins homologous to known effectors, 178 homologous to immune proteins, 446 homologous to core components and 11,673 unannotated proteins. The 519 putative effectors were classified into 20 families (Fig. 5d), comprising 14 putative cargo effector families and 8 putative specialized effector families, with 2 families containing both types (Fig. 5d). Specifically, a total of 87 (16.76%) effectors were identified as potential cargo effectors, with 42 located within an operon with VgrG. The remaining 432 (83.24%) were identified as potential specialized effectors, with the vast majority predicted to be specialized PAAR effectors (427/519, 82.27%). These putative effectors are involved in various functions, and many bacterial polymorphic toxin systems are performed as a toxin module (Fig. 5d and Table S10).

Functional domain annotation of the 87 cargo effectors revealed 26 distinct structural architectures. The most abundant of these are the RHS_core superfamily, followed by DUF6531, which is often associated with Rhs proteins. These modules closely relate to bacterial competition and cellular interactions, playing crucial roles in bacterial virulence factors and secretion systems [76]. The majority of effectors were predicted to be involved in bacterial competition and survival, with structural domains associated with nuclease activity (CdiA-CT, Tox-REase-5, HNHc and FIX_Ntox15_NUC_DUF4112_RhsA-like) [77], lipase activity (EstA and abhydrolase superfamily), bacterial adhesion or invasion (SpaA and ClfA superfamilies) [78,79] and energy metabolism (MenH, Lip2 superfamily and Lipase_3), among others [80,81].

We also performed structural architecture analysis on 432 specialized effectors, revealing 25 distinct structural architectures. The most common was the RHS_core superfamily, while the most frequent toxin domain was the TNT domain, secreted by *Mycobacterium tuberculosis*, which triggers necrosis in infected cells, helping the bacteria evade immune responses [82]. Followed by the Ntox46 superfamily, where proteins containing the Ntox46 domain are secreted through either the type II secretion system or T6SS. This superfamily is found in bacterial polymorphic toxin systems and is known for its role in interbacterial competition. It has been observed in other bacterial groups, including *Pseudomonas* [77]. Another notable domain, Tox-ART-HYD1, was identified in two putative specialized effectors from proteins downstream of *vgrG*. This domain is predicted to function as a toxin within the ADP-ribosyltransferase superfamily, which is present in bacterial polymorphic toxin systems. As reported in Zhang *et al*. [77], proteins containing the Tox-ART-HYD1 were studied in *Bacteroidetes*’s T6SS and function as an effector by modifying host proteins through ADP-ribosylation, disrupting cellular processes in competing microbes or host cells. In particular, proteins containing structural domains such as AHH, EndoU_bacteria and HNHc exhibit nuclease activity in bacteria or viruses. These domains are typically located in the C-terminal region of polymorphic toxin proteins and are primarily associated with bacterial virulence, immune evasion and RNA degradation [83,84]. In addition, Tox-SHH represents the HNH/endonuclease VII fold in bacterial polymorphic toxin systems, characterized by two conserved histidine residues. The toxin is secreted in these systems through type II, type V, type VI or type VII secretion systems [77]. The Tox-MPTase4 is present in polymorphic toxins from phylogenetically diverse bacteria, and in *Escherichia coli*, it is probably released via T6SS [77]. Domains of unknown function, such as DUF3990 and DUF6531, may also play roles in RHS-associated toxicity mechanisms.

Together, these findings underscore the structural and functional diversity of VgrG-associated effectors in *Serratia* and highlight the central role of VgrG in shaping effector repertoires across T6SS loci.

### Comparative genomics and statistical analysis of TAOGs with focus on T6SS^serratia-1b^

**Fig. 6.**
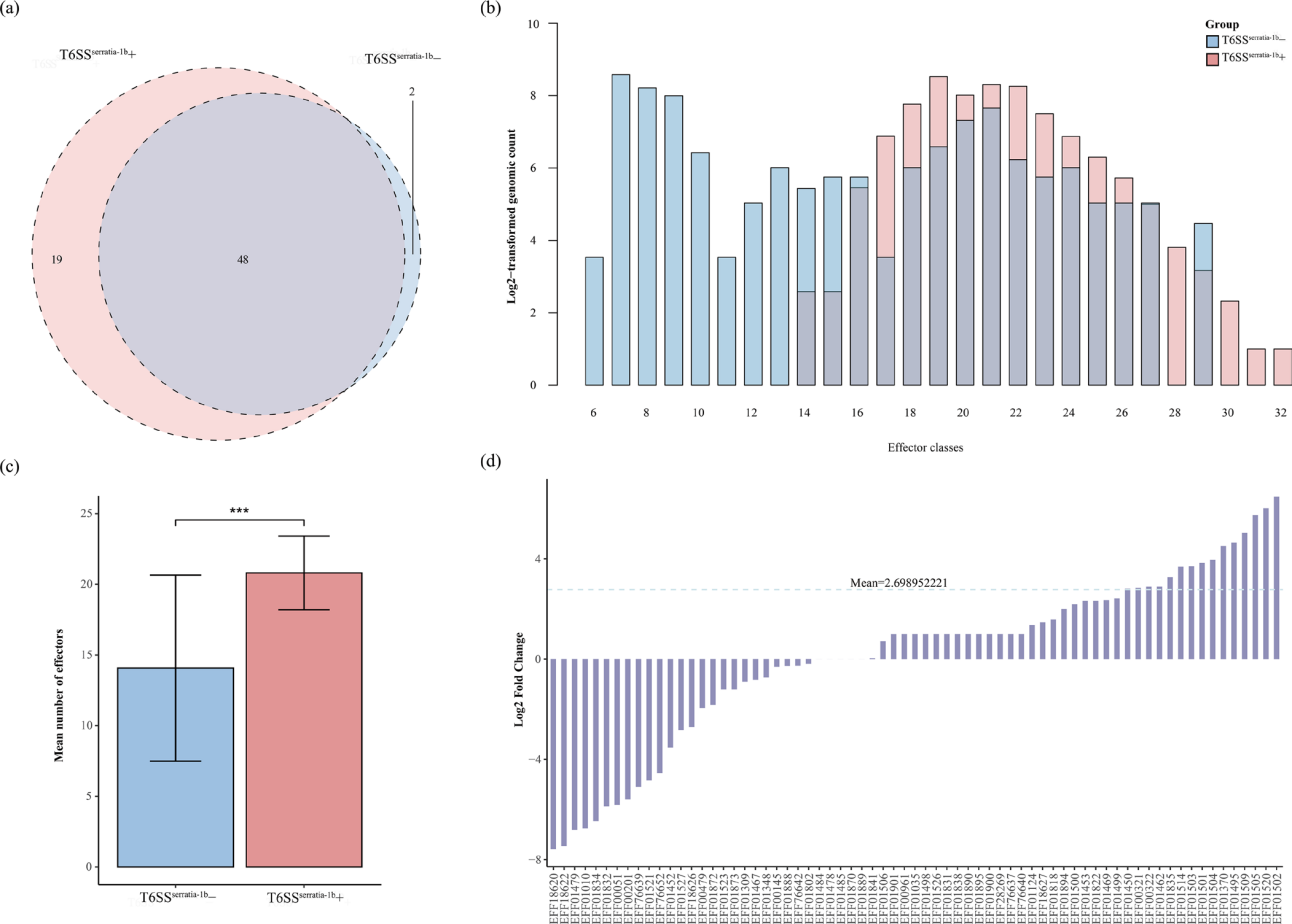
Differences in the distribution of *Serratia* known T6SS-associated proteins in T6SS^serratia-1b^+ and T6SS^serratia-1b^− genomes. (**a**) Venn diagram displays the class numbers of effectors included in the genomes with and without T6SS. (**b**) Histogram displaying the log2-transformed number of genomes relative to the group of effector proteins. The x-axis represents the number of effector proteins, while the y-axis depicts the log2-corrected number of genomes relative to the group of effector proteins. (**c**) Comparison of the mean number of effectors in T6SS^serratia-1b^+ and T6SS^serratia-1b^− genomes using a *t*-test, showing a significantly higher number in T6SS^serratia-1b^+ genomes (***P<0.001). (**d**) Log2-transformed ratio of T6SS^serratia-1b^+ to T6SS^serratia-1b^− genomes for each effector protein group, with the x-axis displaying effector groups and the y-axis indicating fold change.

To investigate the relationship between T6SS-associated protein families and T6SS loci, we employed comparative genomics and statistical methods, thereby identifying their relationship. Statistical analysis was not performed for T6SS^serratia-1a^, T6SS^serratia-2^ and T6SS^serratia-3^ due to their lower distribution in *Serratia*. Consequently, the distribution of known effector, immunity and regulator proteins in the T6SS^serratia-1b^ genome was further discussed. It was observed that the diversity of experimentally validated effectors’ orthologues in T6SS^serratia-1b^+ is higher than T6SS^serratia-1b^− (Fig. 6a). When analysing the distribution of genomes across different effector quantity groups, the counts of T6SS^serratia-1b^ + genomes were predominantly clustered in groups with a higher number of known effectors (Fig. 6b). Furthermore, comparative genome analysis revealed that the number of known effectors in the genomes of T6SS^serratia-1b^+ strains was significantly higher than in those of T6SS^serratia-1b^− strains (*t*-test, *P*<0.001) (Fig. 6c). The known effectors are abundant in T6SS^serratia-1b^+ genomes than T6SS^serratia-1b^− (mean=2.70) (Fig. 6d). Known immunity and regulator proteins also show a similar trend, but the statistical differences of regulatory proteins were less pronounced than those observed in effector and immunity proteins (Figs S3 and S4). In conclusion, the presence or absence of T6SS could be used as an indicator for T6SS-relevant proteins including effectors, immunity proteins and regulators.

**Fig. 7.**
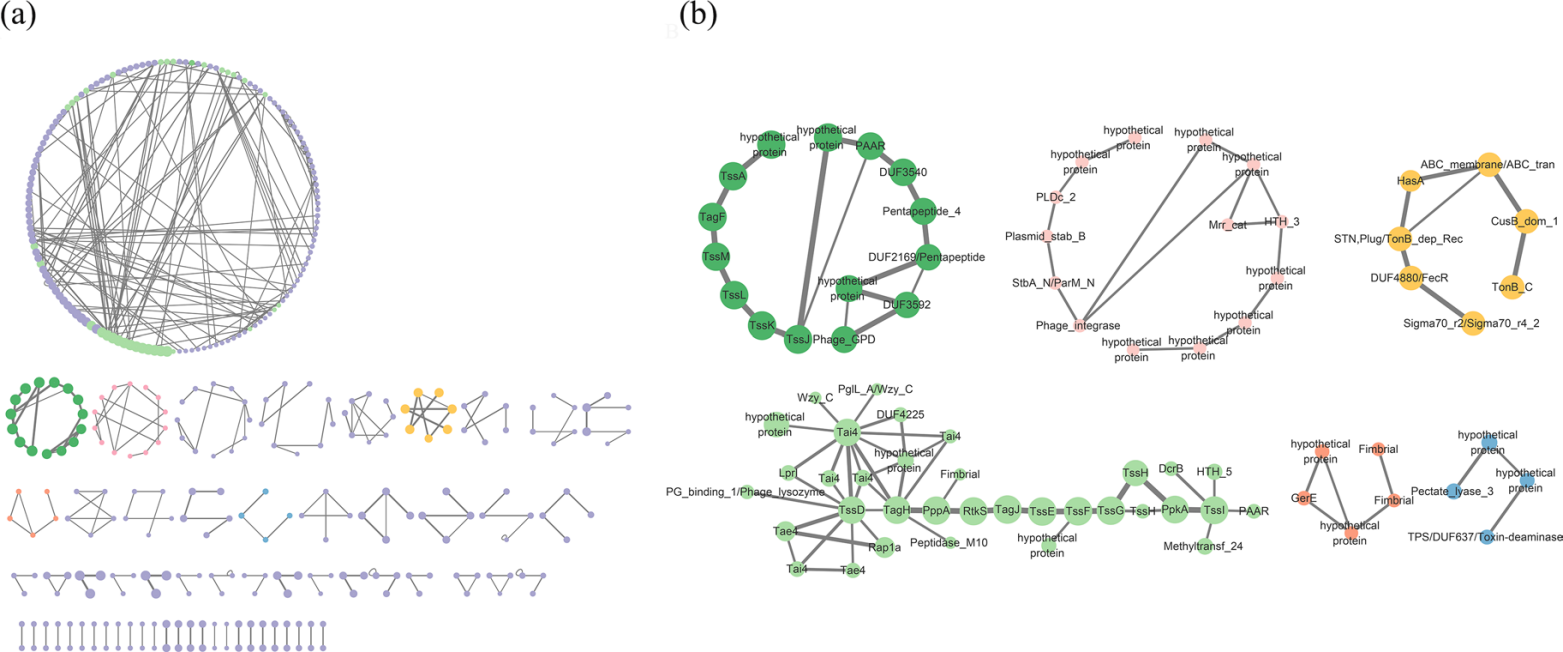
Structural network and distribution of *Serratia* T6SS^serratia-1b^-associated orthologue groups. (**a**) This showed T6SS^serratia-1b^ top 500 protein families (top 500 clusters, log2 presence/absence>3.879, chi-squared test, ***P<0.001). Each node represents a protein orthologous group, and the nodes are connected based on the positions of adjacent genes. The width of the connections between the nodes indicates the number of connections between the two TAOGs, and the size of the nodes is proportional to the number of proteins within each group. (**b**) This is an enlarged view of the corresponding coloured sections from (a), highlighting the connected TAOGs, with a more detailed explanation provided in (b).

Therefore, we have performed a statistical study to explore the orthologues that are significantly associated with T6SS^serratia-1b^ (Fig. 7). Among the top 500 T6SS-relevant protein families, 388 pairs (Tables S11 and S12) and 127 orphan protein families (Table S13) have been identified as TAOGs. Not surprisingly, T6SS^serratia-1b^ was identified among the TAOGs, as 13 of its core components were used as ‘anchors’ to identify other T6SS^serratia-1b^-associated proteins, shown as dark green connected nodes in Fig. 7 (Table S14). Additional proteins not required to define T6SS^serratia-1b^+ and T6SS^serratia-1b^− status but present in the locus, such as TagF, PAAR and DUF2169-containing proteins, were also identified, which demonstrates the effectiveness of our method in detecting both known and novel T6SS-associated components. Interestingly, T6SS^serratia-1a^ was also identified among the TAOGs related to T6SS^serratia-1b^, reinforcing our findings that there is a significant correlation between the presence of T6SS^serratia-1a^ and T6SS^serratia-1b^, which indicates the functional relationship between these two loci. Our analysis identified several T6SS-associated loci and orphan genes that may contribute to genetic diversity and adaptation. For example, proteins containing Phage_integrase, PLDc_2, StbA_N/ParM_N and Plasmid_stab_B domains are known to stabilize plasmids and facilitate genetic exchange [85]. Their presence suggests a potential role in HGT events that could influence the evolution of T6SS-associated functions. Studies on TonB-related proteins in *Salmonella* and *Escherichia* have demonstrated their involvement in iron acquisition [86,87]. Our bioinformatic prediction suggests that in *Serratia*, T6SS may utilize TonB-related proteins for iron acquisition or nutrient transport, functions essential for bacterial survival under competitive conditions. The fimbrial domains play an important role in cell adhesion and biofilm formation [88], potentially contributing to the colonization and persistence in specific ecological niches. Studies have shown that in avian pathogenic *Escherichia coli*, T6SS influences the expression of type I fimbriae and contributes to pathogenesis [89]. Some studies have shown that pili are essential in promoting cell-to-cell contact and facilitating T6SS-mediated competition in bacteria such as *Vibrio cholerae*, *P. aeruginosa* and *Neisseria* [90,92]. We hypothesize that T6SS in *Serratia* may play a role in adhesion and colonization by regulating fimbriae expression. These findings merit further exploration to elucidate the exact roles of these T6SS-associated proteins and their contributions to bacterial fitness, competition and niche adaptation in *Serratia* spp. In addition, 78 clusters of TAOGs, which are predicted as effectors, were not identified either by homology search or position information. Cluster69691 contains the Peptidase_M10 domain, which belongs to the Peptidase_MA family. This enzyme class is known for its ability to cleave peptide bonds, which is crucial in protein hydrolysis [93]. On the other hand, Cluster46067 harbours the Ntox44 domain, which encodes a predicted ribonuclease (RNase) toxin. In bacterial polymorphic toxin systems, Ntox44 is exported through the type II, type VI or type VII secretion systems [77]. These secretion pathways are specialized mechanisms that deliver toxins from bacterial cells to their target cells, enabling the bacteria to exert their toxic effects. Proteins from Cluster85213 and Cluster1327, which contain previously uncharacterized functional domains such as DUF903 and DUF4214, also showed a significant correlation with the presence of T6SS and are worth further exploration.

We selected three putative effector proteins for structural prediction analysis, and the results showed that GCA_000264275.1_01539, GCA_000513215.1_01665 and GCA_000783915.2_00134 (named separately TAOG_01539, TAOG_01665 and TAOG_00134) exhibited structural similarity to the SecReT6 database effectors EFF01889 (ModA), EFF01452 (Rhs-CT5) and EFF01810 (Tlde1), respectively (Fig. S5). TAOG_01539 contains an SBP_bac_1 domain, indicating a potential substrate-binding function [94]. TAOG_01665 contains the RHS domain, Rhs proteins mediate intercellular competition and their C-terminal domains resemble toxic effectors of contact-dependent growth inhibition systems, enabling contact-dependent inhibition of neighbouring cells [95]. TAOG_00134 contains a Tlde1 family domain, classifying it as an antibacterial effector secreted by the T6SS [96]. Additionally, psi-blast results indicate that homologues of these three proteins exist in other bacterial taxa beyond *Serratia* (Fig. S6).

## Discussion

The T6SS serves as a critical environmental adaptation apparatus in bacteria [16]. Despite its ecological and pathogenic relevance, little information is known about the complexity and diversity of T6SS in *Serratia* spp. In this study, we have comprehensively analysed the T6SS clusters in *Serratia* and identified four main subtypes of T6SSs in the *Serratia* genus, characterizing the associated effector, immunity and regulatory proteins, which expanded the database of effector proteins and immunity proteins in *Serratia* spp.

Our findings reveal that T6SS in *Serratia* exhibits both structural diversities, with most species harbouring multiple T6SS loci. Zhang *et al.* [40] reported that, among 116 complete *S. marcescens* genomes, 47 strains (40.5%) contained a single T6SS, while 69 strains (59.5%) harboured 2 or more [40]. In our analysis, out of the 2,337 high-quality genomes, 4.92% (115/2,337) do not contain any loci, 1,378 genomes contain only 1 locus and 35.34% contain multiple loci with the genomes that harbour both T6SS^serratia-1a^ and T6SS^serratia-1b^ the most abundant (756/2,337) (Fig. 2b). This redundancy may reflect the system’s functional versatility, enabling *Serratia* to adapt to specific ecological niches or target different competitors and hosts. Previous studies have elucidated that the T6SS^serratia-1b^ of *S. marcescens* is responsible for antibacterial killing and anti-fungal activity, while its inactivation does not affect the growth rate, motility or biofilm formation [35,67]. Although the functional role of T6SS^serratia-1a^ has not been fully characterized, its strong co-occurrence with T6SS^serratia-1b^ in multiple *Serratia* genomes implies a potential complementary or regulatory function, possibly modulating the efficacy or specificity of T6SS^serratia-1b^-mediated interactions. T6SS^serratia-2^ and T6SS^serratia-3^ are less frequently present in the genus, and their exact functions are unclear.

The four subtypes of T6SS loci presented exhibit high levels of homology across multiple species, and this extensive conservation is typically associated with HGT. These matching strains originate from various sources, with *G. quercinecans* and *Chania multitudinisentens* typically associated with plant and environmental niches [97,98], while *Citrobacter freundii* is often found in environmental and clinical settings [99,100]. This diversity of sources suggests that T6SS loci could have been acquired from environmental bacteria and adapted within *Serratia*, allowing these genes to support interbacterial competition and niche adaptation in diverse environments. Zhang *et al.* [40] reported that the T6SS^serratia-3^ cluster in *S. marcescens* exhibited a substantial similarity to the T6SS of *Klebsiella pneumoniae*. However, our findings suggest a closer similarity to *Citrobacter freundii*. The synteny and sequence alignment analysis comparing the T6SS of *S. marcescens* with the corresponding sequences of T6SS in *K. pneumoniae* and *Citrobacter freundii* revealed that the sequence coverage was 75%, with a similarity of 82.98%. These values were notably higher than the coverage (52%) and similarity (77.82%) observed with *K. pneumoniae*.

In this study, we have not only identified the 11 types of effector families that were characterized in *Serratia*, namely, Ssp1–6, rhs1/rhs2, Slp, Tre1, and Tfe2, but we have also identified 58 types of effector homologous families known from other species (Table S6). Interestingly, the five core shared effector families, which have structural domains belonging to the Tse, Tde, Tae and Tie classes (Fig. 3a), have not been characterized in *Serratia* but have been identified in *A. baumannii*, *Edwardsiella piscicida* and other bacteria, where they function in interbacterial competition.

This study systematically identified T6SS specialized effectors at the genus level in *Serratia* and identified abundant specialized VgrG and PAAR proteins, but no specialized Hcp (Fig. 4 and Tables S7–S9). This may relate to the narrow lumen and C-terminal fusion constraints of Hcp [101] or the presence of unrecognized domains. Analysis of the three genes downstream of *vgrG* identified 519 potential effectors homologous to known proteins, including 432 specialized (mainly PAAR-associated) and 87 cargo effectors, with 42 located in the same operon as VgrG (Table S10). The lower number of cargo effectors may result from the limited and insufficient annotation of related proteins in current SecReT6 database and their high diversity in sequence and structure. While this study expands the cargo effectors, further functional validation is needed. Domain analysis showed that cargo effectors are enriched in functions related to bacterial competition and metabolism. In contrast, specialized effectors are more often associated with virulence, cytotoxicity and immune evasion (Fig. 5). These findings expand the T6SS effector repertoire in *Serratia* and highlight the functional diversity and ecological roles of these effector proteins.

Considering the current limitations of identifying T6SS effectors by traditional experimental means, we employed a comparative genomic approach that does not rely on traditional sequence homology or *vgrG* downstream gene location lookup, enabling the broad identification of not only T6SS effectors but also other T6SS-associated genes. We observed that several domains, such as Phage_GPD and Phage_integrase, are present among the TAOGs, which supports the hypothesis that the T6SS may have evolved through a bacteriophage-like mechanism [30], sharing structural or functional similarities with phage assembly machinery [102]. It was reported that some types of T6SS loci also functionally participated in nutrient acquisition [103,104]. We found that membrane-related proteins, including components of the TonB complex and ABC transporters, were also frequently associated with T6SS^serratia-1b^. These proteins are critical for nutrient acquisition, especially iron, essential for bacterial survival in nutrient-limited environments. The involvement of TonB-dependent receptors suggests that T6SS in *Serratia* may be involved in resource acquisition and allowing bacteria to compete more effectively in iron-limited niches, such as within host organisms. These TAOGs highlight the complex regulatory and functional diversity of T6SS in *Serratia*, and further experimental validation is required to confirm the functions and fully understand their contributions to T6SS-mediated interactions.

While our comparative genomic approach offers a powerful means to identify TAOGs across a large number of genomes, we acknowledge certain limitations. First, the presence/absence associations identified are correlative and do not imply direct functional involvement in T6SS activity. Some co-occurring gene families may reflect shared evolutionary history or genomic co-localization due to HGT, rather than a mechanistic link to T6SS function. Second, the reliance on *in silico* predictions, including homology-based and domain-based classifications, cannot substitute for experimental validation. Future studies involving transcriptomic, proteomic and mutational analyses will be essential to confirm the roles of these candidate proteins in T6SS-related processes.

In conclusion, our study extended our knowledge of T6SS in *Serratia*, highlighting its significance in bacterial ecology and competition. The high degree of protein diversity and evidence of HGT suggest that *Serratia*’s T6SS is an adaptable system that may respond actively in response to environmental stresses. Future studies experimentally validate the functions and roles of T6SS-associated proteins identified here and explore their roles in pathogenicity and host interactions.

## Supplementary material

10.1099/mgen.0.001424 Uncited Supplementary Material 1.

10.1099/mgen.0.001424 Uncited Supplementary Material 2.

## Abbreviations

ANI: average nucleotide identity
Hcp: haemolysin-coregulated protein
HGT: horizontal gene transfer
HMM: hidden Markov model
PAAR: proline−alanine−alanine−arginine
psi-blast: Position-Specific Iterated blast
TAOG: T6SS-associated orthologous group
T6SS: type VI secretion system
VgrG: valine−glycine repeat protein G
