# Supplementary material for: Comprehensive genomic analysis of type VI secretion system diversity and associated proteins in Serratia
Source: Microb Genom. 2025 Jun 13;11(6):001424. doi: 10.1099/mgen.0.001424 (PMC12165300; doi:10.1099/mgen.0.001424)
Supplement: Uncited Supplementary Material 1. [file mgen-11-01424-s001.pdf]

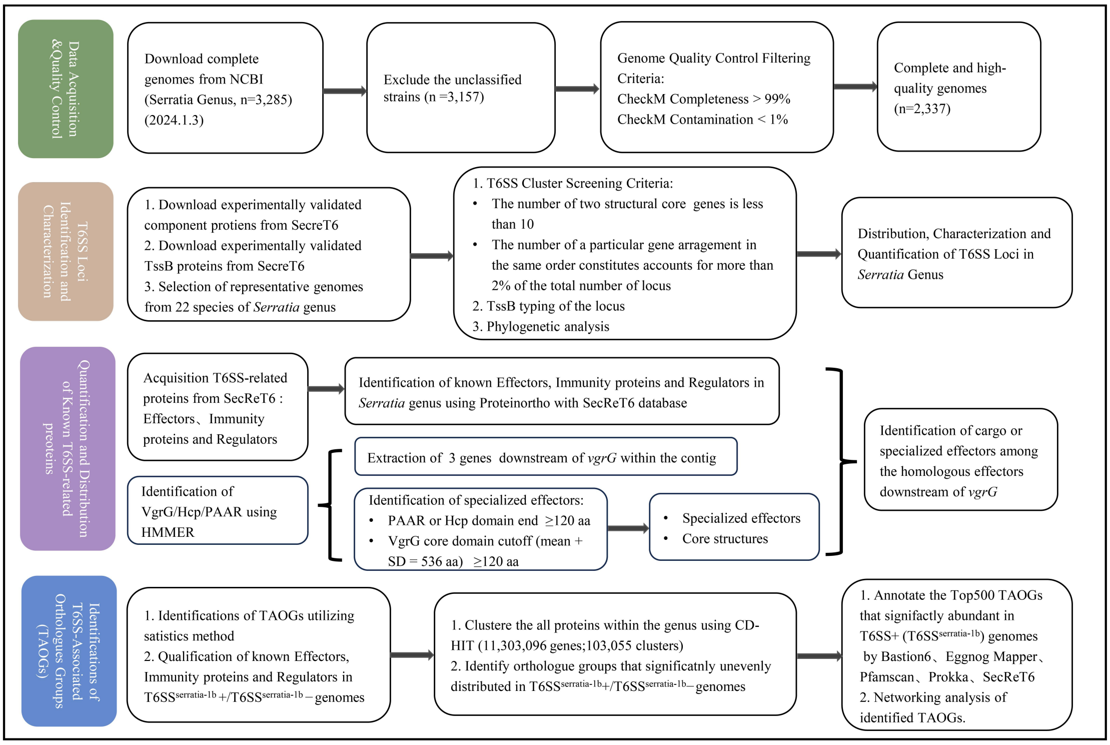

Fig. S1 Workflow overview of T6SS analysis in *Serratia* spp.. Illustrating the sequential steps for data acquisition, T6SS loci characterization, identification of known T6SS-relevant proteins, exploration of specialized effectors, and discovery of T6SS-associated orthologous groups.

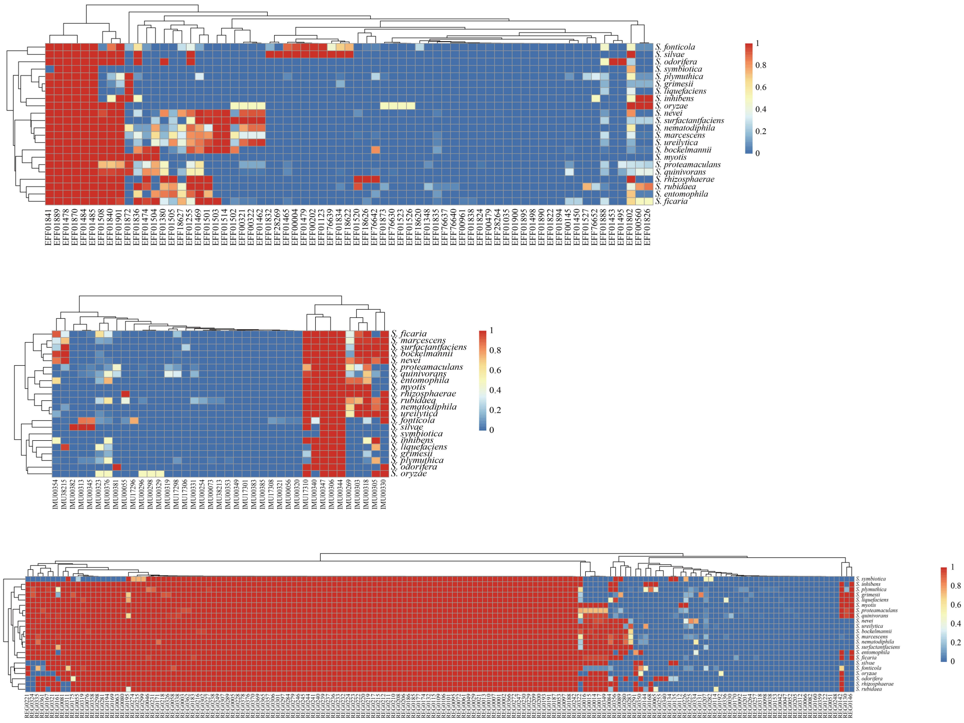

Fig. S2 Homology comparison and distribution of known T6SS-associated proteins in *Serratia*. The rows represent 22 *Serratia* species, and the column labels indicate known T6SS effectors, immunity, and regulator protein families.

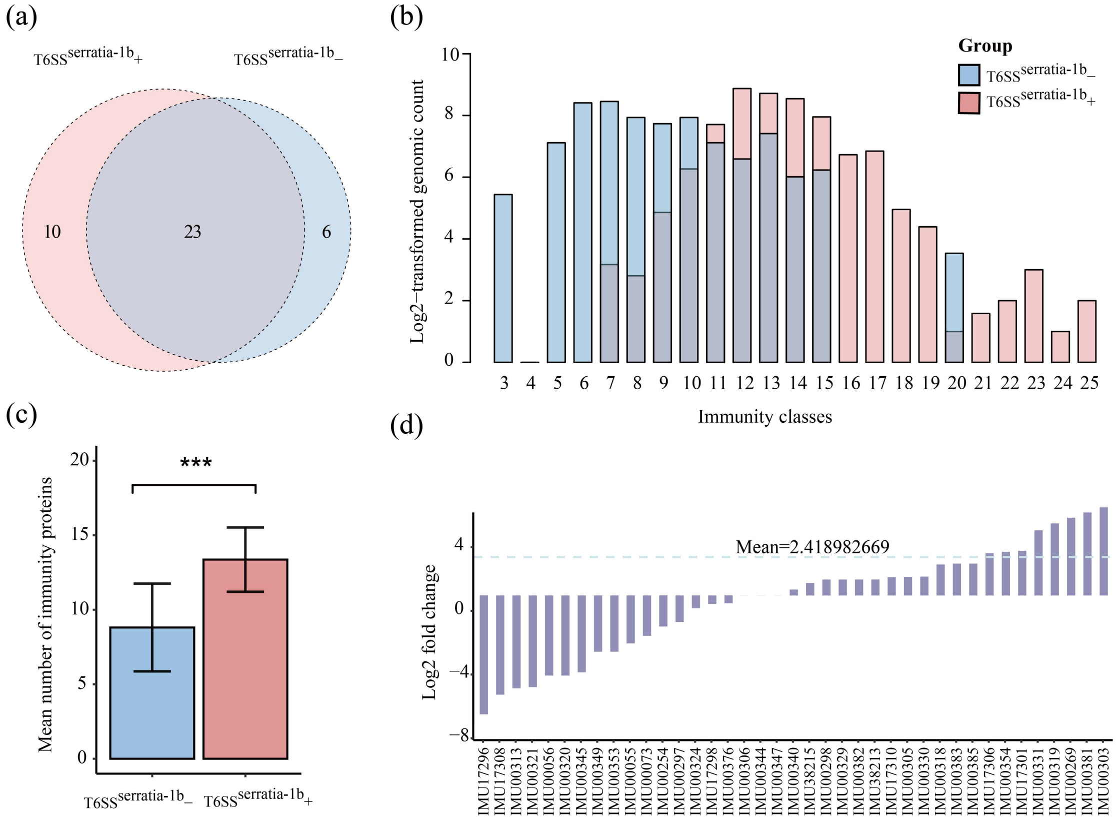

Fig. S3 Differences in the distribution of *Serratia* known T6SS immunity proteins in T6SS$^{\text{serratia-1b}}$+ and T6SS$^{\text{serratia-1b}}$– genomes. (a) Venn diagram displays the class numbers of immunity proteins included in the genomes with and without T6SS. (b) Histogram displaying the log2-corrected number of genomes relative to the group of immunity proteins. The the number of immunity proteins, while the Y-axis depicts the log2-corrected number of genomes relative to the group of immunity proteins. (c) Comparison of the mean number of immunity proteins in T6SS$^{\text{serratia-1b}}$+ and T6SS$^{\text{serratia-1b}}$– genomes using a *t*-test, showing a significantly higher number in T6SS$^{\text{serratia-1b}}$+ genomes (***, $p < 0.001$). (d) Log2-transformed ratio of T6SS$^{\text{serratia-1b}}$+ to T6SS$^{\text{serratia-1b}}$– genomes for each immunity protein group, with the X-axis displaying immunity proteins groups and the Y-axis indicating fold change.

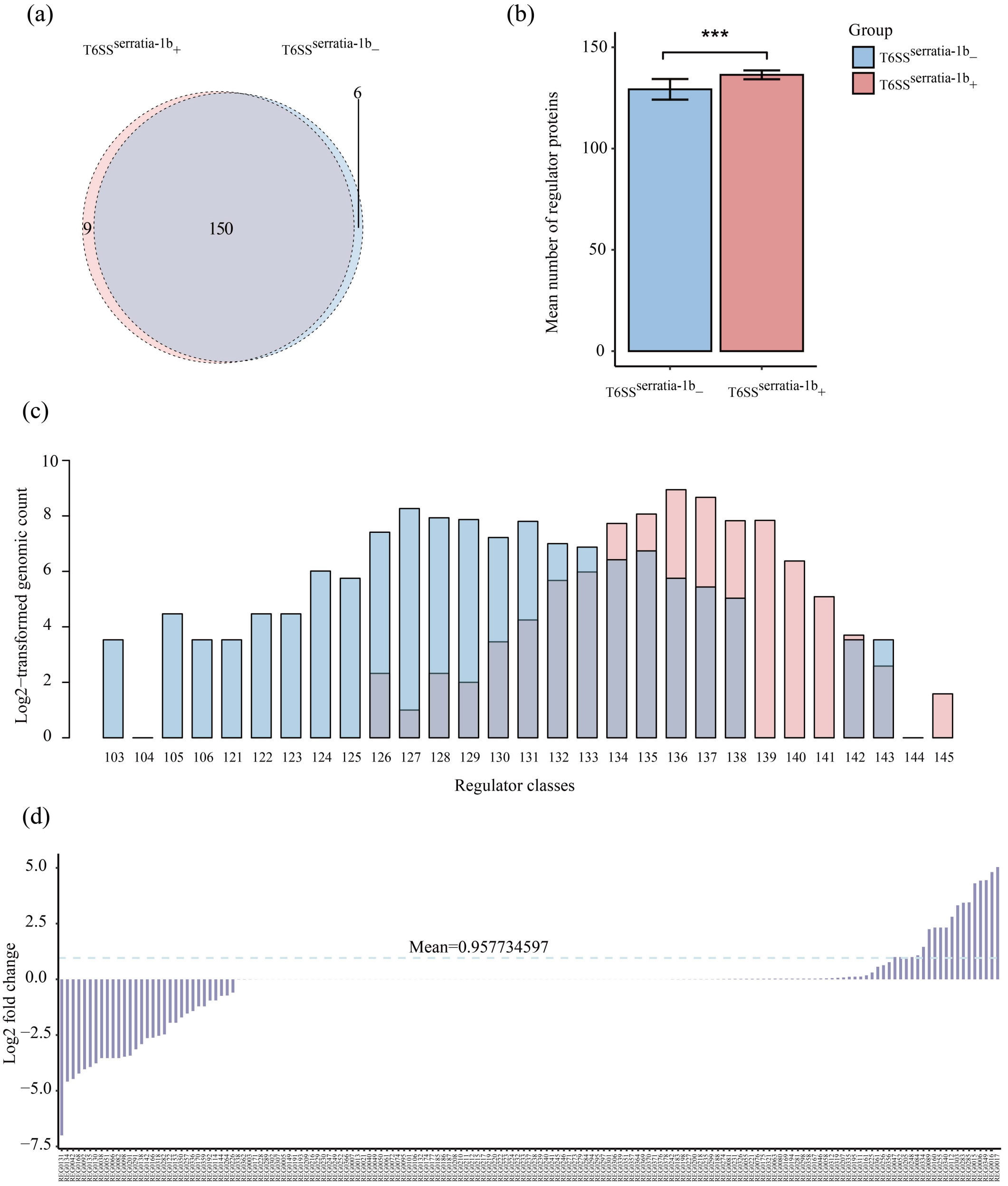

Fig. S4 Differences in the distribution of *Serratia* known T6SS regulator proteins in T6SS$^{\text{serratia-1b}}$+ and T6SS$^{\text{serratia-1b}}$– genomes. (a) Venn diagram displays the class numbers of regulator proteins included in the genomes with and without T6SS. (b) Comparison of the mean number of regulator proteins in T6SS$^{\text{serratia-1b}}$+ and T6SS$^{\text{serratia-1b}}$– genomes using a *t*-test, showing a significantly higher number in T6SS$^{\text{serratia-1b}}$+ genomes (***, $p < 0.001$). (c) Histogram displaying the log2-corrected number of genomes relative to the group of regulator proteins. The X-axis represents the number of regulator proteins, while the Y-axis depicts the log2-corrected number of genomes relative to the group of regulator proteins. (d) Log2-transformed ratio of T6SS$^{\text{serratia-1b}}$+ to T6SS$^{\text{serratia-1b}}$– genomes for each regulator protein group, with the X-axis displaying regulator proteins groups and the Y-axis indicating fold change.

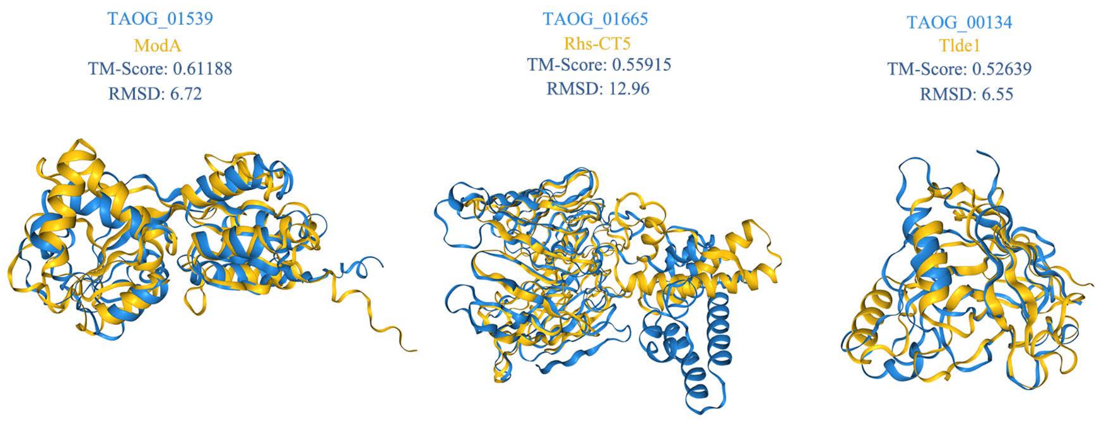

Fig. S5 The structural predictions of three predicted effectors. Their three-dimensional structures were predicted using AlphaFold2 and compared for structural similarity with SecReT6 effectors using Foldseek. The yellow structures represent the known SecReT6 effectors, while the blue structures represent the three potential effectors predicted in this study. The overlayed regions indicate structural similarity between the two. TM-score and Root Mean Square Deviation (RMSD) from AlphaFold2 predictions were used to assess structural similarity**.**

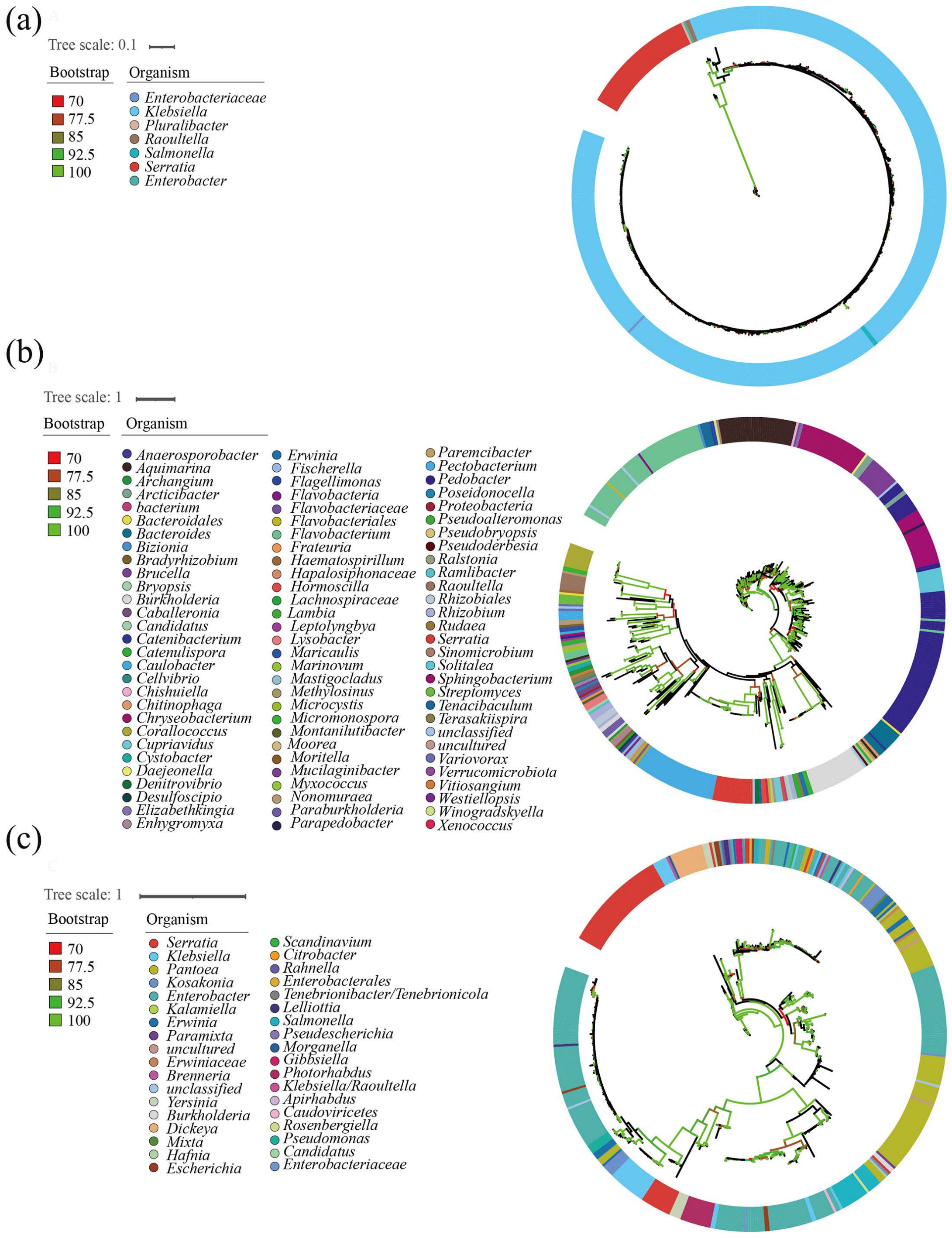

Fig. S6 Phylogenetic trees of three putative T6SS effector proteins based on PSI-BLAST results. Each protein sequence: GCA_000264275.1_01539 (a), GCA_000513215.1_01665 (b), and GCA_000783915.2_00134 (c) was queried against the NCBI nr database using psi-blast (three iterations, E-value cutoff of 0.001), and the top 500 homologous sequences were used to construct maximum likelihood phylogenetic trees. Bootstrap values are color-coded, and the outer rings indicate the taxonomic classification of each hit.
